# Porcine circovirus type 2 (PCV2) infection decreases the efficacy of an attenuated classical swine fever virus (CSFV) vaccine

**DOI:** 10.1186/1297-9716-42-115

**Published:** 2011-12-01

**Authors:** Yu-Liang Huang, Victor Fei Pang, Chun-Ming Lin, Yi-Chieh Tsai, Mi-Yuan Chia, Ming-Chung Deng, Chia-Yi Chang, Chian-Ren Jeng

**Affiliations:** 1 Graduate Institute of Veterinary Medicine, School of Veterinary Medicine, National Taiwan University, No. 1, Sec. 4, Roosevelt Rd., Taipei 106, Taiwan; 2 Division of Hog Cholera Research, Animal Health Research Institute, Council of Agriculture, No. 376, Chung-Cheng Rd., Tansui District, New Taipei City 251, Taiwan; 3 Graduate Institute of Molecular and Comparative Pathobiology, School of Veterinary Medicine, National Taiwan University, No. 1, Sec. 4, Roosevelt Rd., Taipei 106, Taiwan

## Abstract

The Lapinized Philippines Coronel (LPC) vaccine, an attenuated strain of classical swine fever virus (CSFV), is an important tool for the prevention and control of CSFV infection and is widely and routinely used in most CSF endemic areas, including Taiwan. The aim of this study was to investigate whether PCV2 infection affects the efficacy of the LPC vaccine. Eighteen 6-week-old, cesarean-derived and colostrum-deprived (CDCD), crossbred pigs were randomly assigned to four groups. A total of 10^5.3 ^TCID_50 _of PCV2 was experimentally inoculated into pigs through both intranasal and intramuscular routes at 0 days post-inoculation (dpi) followed by LPC vaccination 12 days later. All the animals were challenged with wild-type CSFV (ALD stain) at 27 dpi and euthanized at 45 dpi. Following CSFV challenge, the LPC-vaccinated pigs pre-inoculated with PCV2 showed transient fever, viremia, and viral shedding in the saliva and feces. The number of IgM^+^, CD4^+^CD8^-^CD25^+^, CD4^+^CD8^+^CD25^+^, and CD4^-^CD8^+^CD25^+ ^lymphocyte subsets and the level of neutralizing antibodies against CSFV were significantly higher in the animals with LPC vaccination alone than in the pigs with PCV2 inoculation/LPC vaccination. In addition, PCV2-derived inhibition of the CSFV-specific cell proliferative response of peripheral blood mononuclear cells (PBMCs) was demonstrated in an ex vivo experiment. These findings indicate that PCV2 infection decreases the efficacy of the LPC vaccine. This PCV2-derived interference may not only allow the invasion of wild-type CSFV in pig farms but also increases the difficulty of CSF prevention and control in CSF endemic areas.

## Introduction

Classical swine fever virus (CSFV) is an enveloped, positive sense, and single stranded RNA virus belonging to the genus *Pestivirus *within the family *Flaviviridiae *[1,2]. This virus causes systemic hemorrhage in domestic pigs and leads to severe economic losses in the swine industry worldwide. Currently, classical swine fever (CSF) is still rampant in most Asian and Latin American and in some European countries [1].

In most endemic areas, such as Taiwan, Lapinized Philippines Coronel (LPC) vaccine, an attenuated CSFV strain, is an important tool for prevention and control of CSF. The LPC is attenuated from a virulent CSFV strain by several hundred passages in the rabbit and is used as a modified live CSFV vaccine [3]. The LPC vaccine is able to induce complete protection in pigs against virulent CSFV challenge, and LPC-vaccinated pigs do not show any clinical signs, viremia or shedding of CSFV [4,5].

Porcine circovirus type 2 (PCV2) is distributed worldwide and has been suggested as a major causative agent of postweaning multisystemic wasting syndrome (PMWS) in pigs [6,7]. The characteristics of clinical and immunological pathology in PMWS-affected pigs are lymphocyte depletion, increase in population of monocyte/macrophage lineage cells in lymphoid tissues and peripheral blood, and irregular cytokine responses constitutionally or after stimulation [7,8]. It is known that PCV2 infection decreases the protection efficacy of modified porcine reproductive and respiratory syndrome virus (PRRSV) vaccine [9]. In addition, PCV2 infection could immunomodulate the pseudorabies virus (PRV) recall antigen responses in pigs, either by viral components or through soluble factors like IL-10 [10,11]. Therefore, the aim of this study was to investigate whether PCV2 infection could decrease or interfere with the efficacy of LPC vaccine.

PMWS primarily occurs in pigs between 25 and 120 days of age, with high prevalence between 60 and 80 days of age [7]. Unfortunately, the susceptible age of PCV2 infection in pigs overlaps with the age of LPC vaccination, which is around the postweaning stage in the management of the pig farm [4,7]. This situation highlights the importance of this study in the area using LPC vaccine to control CSF. In the present study, experimentally PCV2-infected and LPC-vaccinated cesarean-derived, colostrum-deprived (CDCD) piglets were challenged with wild-type CSFV. Clinical signs, viremia, viral shedding, change in lymphocyte subpopulations, as well as humoral and cell-mediated immune (CMI) responses were evaluated. It was revealed that PCV2 infection could decrease the efficacy of LPC vaccine and the mechanisms of the immune interference were also demonstrated.

## Materials and methods

### Virus and cells

A PCV1-free porcine kidney cell line (PK-15) was used to propagate PCV2 and CSFV (ALD strain) [12]. The PCV2 was isolated from a PMWS-affected pig and it was PCV2b in its genotype by sequencing. The ALD is a high virulent strain of CSFV isolated from Japan that it has been demonstrated to cause severe clinical signs and lesions in infected pigs [12] and is a challenged strain for evaluating the efficacy of LPC vaccine in Taiwan. The viral titers of PCV2 and the ALD strain of CSFV were determined to be 10^5.0 ^and 10^6.8 ^TCID_50 _per mL, respectively, by virus titration [1].

### Experimental animals

The animal experiments performed in the present study were all approved by the Institutional Animal Care and Use Committee of Animal Health Research Institute (98013) under the guidelines of the Animal Protection Act in Taiwan. Eighteen 6-week-old, crossbred CDCD pigs were randomly divided into four groups and housed in four separated biocontainment animal rooms at the Animal Health Research Institute as shown in Table 1. The pigs in groups 1 and 2 were intranasally inoculated with 0.5 mL of PCV2 in each nostril and were intramuscularly injected with 1 mL of PCV2 at the right side of the dorsal neck region at 0 dpi (a total dose of 10^5.3 ^TCID_50 _per pig). The pigs in groups 1 and 3 were intramuscularly injected with 1 dose of LPC vaccine/pig at the right side of the dorsal neck region at 12 dpi. Due to that pigs obtained complete protection against virulent CSFV challenge as early as 1 week after LPC vaccination and the neutralizing antibodies in LPC-vaccinated pigs were detectable at 2-3 post-vaccination weeks [4], a 15-day-duration between vaccination and challenge was selected as a proper time frame to see the interference of vaccine efficacy by PCV2 infection. Therefore, all pigs were challenged with 1 mL of CSFV (ALD strain)/pig by intramuscular injection at the left side of the dorsal neck region at 27 dpi (a total dose of 10^6.8 ^TCID_50 _per pig). Clinical monitoring was recorded daily, and rectal temperature and sample collection of blood, saliva, and feces were taken every 3 days until 45 dpi. All surviving pigs were necropsed at 45 dpi.

**Table 1:** Experimental design^a^.

Group number	Pig number	PCV2	LPC	CSFV
Group 1	6	+	+	+
Group 2	6	+	-	+
Group 3	3	-	+	+
Group 4	3	-	-	+

PCV2: Porcine circovirus type 2; LPC: Lapinized Philippines Coronel; CSFV: Classical swine fever virus.

Group 1: PCV2-infected/LPC-vaccinated/CSFV-challenged; group 2: PCV2-infected/CSFV-challenged; group 3: LPC-vaccinated/CSFV-challenged, and group 4: CSFV-challenged.

^a^The pigs were inoculated with 10^5.3 ^TCID_50 _of PCV2 (group 1 and 2), 1 dose of LPC vaccine (group 1 and 3), and 10^6.8 ^TCID_50 _of wild-type CSFV (ALD) (group 1, 2, 3, and 4) at 0, 12, and 27 dpi, respectively.

### Detection of PCV2 and CSFV viremia and shedding

PCV2 and CSFV in plasma, saliva, and feces were detected by real-time PCR (rPCR) or reverse transcription real time PCR (rRT-PCR), respectively. Total nuclear acid of plasma, saliva, and feces was extracted by MegNA Pure LC Total Nucleic Acid Isolation kit according to the manufacturer's instructions (Roche, Mannheim, Germany). Universal-PCV primers which were the common primers for PCV1, PCV2a, and PCV2b and specific-PCV2 TaqMan probe were used in rPCR for PCV2 detection. The primer and probe sequences were 5'-GCTGGCTGAACTTTTGAAA-3', 5'-CCTTTAGTCTCTACAGTCAATGGAT-3', and 5'-FAM-TGCTAATTTTGCAGACCCGGAAACCAC-BHQ1-3'. The rPCR of PCV2 was carried out in the LightCycler^® ^480 system (Roche, Mannheim, Germany) in a reaction volume of 20 μL, containing 5 μL of DNA, 1× LightCycler^® ^TaqMan^® ^Master (Roche, Mannheim, Germany), 0.15 μM PCV2 TaqMan probe, and 0.5 μM of each universal-PCV primer. The reaction condition involved initial incubation at 95°C for 10 min followed by 45 cycles of denaturation (95°C for 20 s), annealing (60°C for 30 s), and extension (72°C for 20 s). The rRT-PCR for CSFV detection was the same as that described by Huang et al. [1].

### Analysis of lymphocyte subpopulation change in peripheral blood

The total number of lymphocytes in peripheral blood was counted by MS4-PACK^® ^kit according to the manufacturer's instructions (Melet Schloesing Laboratories, Osny, France) and they were further divided into IgM^+^, CD4^-^CD8^+^CD25^+^, CD4^+^CD8^-^CD25^+^, and CD4^+^CD8^+^CD25^+ ^subgroups by flow cytometry using monoclonal antibodies (mAbs) against IgM, CD4, CD8, and CD25 surface antigens on porcine lymphocytes. Briefly, lymphocytes were isolated by the Uti-Lyse kit according to the manufacturer's instructions (Dakocytomation, Carpinteria, CA, USA) and were divided into the IgM subgroup and the CD4CD8CD25 subgroup. The lymphocytes in the IgM subgroup were stained with mouse anti-pig IgM mAb (AbD Serotec, Kidlington, UK), followed by FITC-conjugated rat anti-mouse IgG1 mAb (AbD Serotec, Kidlington, UK). The lymphocytes in the CD4CD8CD25 subgroup were stained with FITC-conjugated mouse anti-pig CD4 mAb, PE-conjugated mouse anti-pig CD8 mAb (BD Biosciences, Sunnyvale, CA, USA), and mouse anti-pig CD25 mAb (AbD Serotec, Kidlington, UK), followed by PerCP-conjugated rat anti-mouse IgG1 mAb (BD Biosciences, Sunnyvale, CA, USA), which can link mouse anti-pig CD25 mAb. Finally, lymphocytes in each sample were gated and analyzed by CellQuest software from FACSCalibur cytometry (BD Biosciences, Sunnyvale, CA, USA). The absolute number of each subgroup was calculated as follows: absolute number = total lymphocytes × percentage of positive cells on lymphocyte gate.

### Detection of PCV2 antibody and CSFVneutralizing antibody

PCV2 antibody in serum was detected by SERELISA PCV2 Ab Mono Blocking kit according to the manufacturer's instructions (Synbiotics Europe SAS, Lyon Cedex, France). The neutralizing antibody of CSFV in serum was detected using the OIE protocol [2].

### Ex vivo CSFV-specific cell proliferative response of PBMCs

To understand how the immunomodulation of PCV2 infection affecting the efficacy of LPC vaccine, an ex vivo CSFV-specific cell proliferative response of PBMCs was performed. Because the CSFV-specific cell proliferative response of PBMCs can only be detected in pigs 30 days after virulent CSFV infection [13], another animal experiment was established to perform this assay. Briefly, five 4-week-old, non-vaccinated, healthy, conventional piglets were obtained from a commercial herd without the history of specific swine diseases, including CSF, PRRS, swine influenza, and pseudorabies. The piglets were negative in antibody and nuclear acid detection of the four viral pathogens mentioned above by RT-PCR or PCR and commercial ELISA kits, but PCV2 was detected by rPCR with the amount of 10^1.2 ^to 10^3.3 ^copies of PCV2 genome per 10^6 ^PBMCs. The piglets were housed in a biocontainment animal house at the Animal Health Research Institute, where they were vaccinated with LPC vaccine at the right side of the dorsal neck region at 4 and 7 weeks of age and inoculated with 1 mL of CSFV (ALD strain)/pig by intramuscular injection at the left side of the dorsal neck region at 9 weeks of age (a total dose of 10^6.8 ^TCID_50 _per pig). Each pig was bled at 15-17 weeks of age and K_3_EDTA (Sigma-Aldrich, St. Louis, MO, USA) was used to prevent coagulation. The animal experiments performed in the present study were all approved by the Institutional Animal Care and Use Committee of Animal Health Research Institute (97013) under the guidelines of the Animal Protection Act in Taiwan.

PBMCs were isolated from 8 mL of anti-coagulated blood with K_3_EDTA by centrifugation at 400 × *g *on Histopaque-1077 (Sigma-Aldrich, St. Louis, MO, USA). PBMCs at 10^6 ^cells/mL were cultured in RPMI-1640 (Invitrogen, Carlsbad, CA, USA) containing 10% (vol/vol) heat-inactivated fetal bovine serum, 100 units/mL penicillin G, 100 μg/mL streptomycin, and 0.25 μg/mL amphotericin B. All of the fetal bovine serum used in the present study tested negative for pestivirus by RT-PCR and anti-pestivirus antibodies by IFA.

To evaluate whether PCV2 has an effect on the CSFV-specific cell proliferative response of PBMCs, the 96-well plates with seeded PBMCs at 10^5 ^cells/well were classified into a mock group, ALD group, PCV2-0.1/ALD group, PCV2-0.05/ALD group, PCV2-0.01/ALD group, UV-inactivated PCV2-0.1/ALD, and Con A group. PCV2 at 0.1, 0.05, and 0.01 MOI and 0.1 MOI of UV-inactivated PCV2 were separately added into PBMCs of each group 18 h before stimulation with 1 MOI of ALD. Concanavalin A at 5 μg/mL (Sigma-Aldrich, St. Louis, MO, USA) was added into the Con A group as the control. Following CSFV or Con A stimulation, the proliferative responses of PBMCs were measured by cell proliferation ELISA kit (Roche, Mannheim, Germany) at 4 days post-stimulation (dps). The kit detects bromodeoxyuridine (BrdU) incorporation during DNA synthesis in proliferating cells. The assay procedure for PBMC proliferative response was performed according to the manufacturer's instructions.

### Statistical analysis

The ratio of feverish, viremia, and viral shedding animals was analyzed by the Fisher Exact Probability test. Comparisons of the values between two groups and among various groups were analyzed by the Student's *t*-test and one-way analysis of variance (ANOVA), respectively. ANOVA was combined with the Duncan multiple range test. The statistical analysis of the data was carried out by statistical analysis system (Statistical Analysis System; SAS for Windows 6.12; SAS Institute Inc., Cary, NC, USA). A *P *value of less than 0.05 was considered to be statistically significant.

## Results

### PCV2 infection caused transient fever and clinical signs in LPC-vaccinated animals after CSFV challenge

Clinically, all of the pigs with LPC vaccination (group 1 and group 3) survived and the majority of pigs in both groups showed no fever or clinical syndromes such as depression, anorexia, and cyanosis. Transient fever (> 40.5°C) was noted in one of the animals previously inoculated with PCV2 (group 1) during the 6^th ^to 12^th ^day after challenge (Table 2). Although no statistical significant difference in the number of the feverish animals in both groups was noted, the duration of fever in group 1 (1.5 ± 1.5 days) was significantly longer than that of group 3 (0 ± 0 days) and the average rectal temperature in group 1 was significantly higher than that of group 3 from the 3^rd ^to the 9^th ^day after wild-type CSFV challenge. In the pigs without LPC vaccination, all animals showed clinical signs and fever. The animals started to die at the 12^th ^day after challenge and all the pigs died at the 16^th ^day post challenge. Compared to the simple CSFV infection (group 4), the average survival days of pre-infection with PCV2 and then a challenge with wild-type CSFV (group 2) were significantly shorter than that of group 4 (13.8 ± 1.3 vs. 15.7 ± 0.5 days).

**Table 2:** Changes in the number of pigs developing fever in various treatment groups over time after classical swine fever virus (CSFV) challenge^a^.

dpi	27	30	33	36	39	42	45
Group 1	0/6^b^	0/6	1/6	1/6	1/6	0/6	0/6
Group 2	0/6	6/6	3/6	4/6	3/4	0/1	0/0
Group 3	0/3	0/3	0/3	0/3	0/3	0/3	0/3
Group 4	0/3	1/3	2/3	2/3	2/3	1/3	0/0

Group 1: PCV2-infected/LPC-vaccinated/CSFV-challenged; group 2: PCV2-infected/CSFV-challenged; group 3: LPC-vaccinated/CSFV-challenged, and group 4: CSFV-challenged.

^a^The pigs were inoculated with 10^5.3 ^TCID_50 _of PCV2 (group 1 and 2), 1 dose of LPC vaccine (group 1 and 3), and 10^6.8 ^TCID_50 _of wild-type CSFV (ALD) (group 1, 2, 3, and 4) at 0, 12, and 27 dpi, respectively.

^b^Number of animals with fever/number of surviving animals.

### PCV2 infection caused transient wild-type CSFV viremia and viral shedding in saliva and feces in LPC-vaccinated animals after CSFV challenge

Viremia and viral shedding of the challenged wild-type virus in the vaccinated animals were important factors by which to evaluate the efficacy of vaccination. In this study, no viremia and viral shedding of wild-type CSFV were noted in the experimental animals with LPC vaccination alone (group 3) (Tables 3, 4, and 5). Transient viremia and viral shedding of wild-type CSFV in the saliva and feces were noted in the animals previously infected with PCV2 (group 1). The viremia first appeared at the 3^rd ^day after challenge and could last up to the 15^th ^day post-challenge (Table 3). The appearance of viral shedding in the saliva was slower and the duration was shorter, which could only be detected at 9 to 12 days post-challenge (Table 4). Fecal viral shedding had a similar pattern and was also detected only between the 9^th ^and 15^th ^day post-challenge (Table 5). Following wild-type CSFV challenge, there were only one to three out of 6 pigs in group 1 developing viremia or viral shedding (Tables 3, 4, and 5). Although no statistical significant difference in the number of the viremia and shedding animals of wild-type CSFV in both groups was noted, the duration of viremia (3.5 ± 3.6 days) and viral shedding (2 ± 1.4 days in saliva and 3 ± 1.7 days in feces) in group 1 was significantly longer than that of group 3 (0 ± 0 days in viremia, saliva, and feces). In the wild-type CSFV-challenged animals without LPC vaccination of groups 2 and 4, viremia and viral shedding could be detected more profoundly (Tables 3, 4, and 5). Viremia and viral shedding of PCV2 were detected in the two PCV2-inoculated groups and groups 1 and 2; however, there were no significant differences regarding the frequency and duration between the two groups (Tables 3, 4, and 5).

**Table 3:** Changes in the number of pigs developing porcine circovirus type 2 (PCV2) and wild-type classical swine fever virus (CSFV) viremia in various treatment groups over time after CSFV challenge^a^.

dpi	27	30	33	36	39	42	45
Group 1	6/0/6^b^	6/3/6	6/2/6	6/1/6	6/1/6	6/1/6	6/0/6
Group 2	6/0/6	6/6/6	6/6/6	6/6/6	4/4/4	1/1/1	0/0/0
Group 3	0/0/3	0/0/3	0/0/3	0/0/3	0/0/3	0/0/3	0/0/3
Group 4	0/0/3	0/3/3	0/3/3	0/3/3	0/3/3	0/3/3	0/0/0

Group 1: PCV2-infected/LPC-vaccinated/CSFV-challenged; group 2: PCV2-infected/CSFV-challenged; group 3: LPC-vaccinated/CSFV-challenged, and group 4: CSFV-challenged.

^a^The pigs were inoculated with 10^5.3 ^TCID_50 _of PCV2 (group 1 and 2), 1 dose of LPC vaccine (group 1 and 3), and 10^6.8 ^TCID_50 _of wild-type CSFV (ALD) (group 1, 2, 3, and 4) at 0, 12, and 27 dpi, respectively. The PCV2 and wild-type CSFV loads in plasma were detected by real-time PCR or reverse transcription real-time PCR, respectively.

^b^Number of PCV2-positive animals/number of wild-type CSFV-positive animals/number of surviving animals.

**Table 4:** Changes in the number of pigs showing viral shedding of porcine circovirus type 2 (PCV2) and wild-type classical swine fever virus (CSFV) in the saliva of various treatment group over time after CSFV challenge^a^.

dpi	27	30	33	36	39	42	45
Group 1	6/0/6^b^	4/0/6	4/0/6	5/2/6	6/2/6	6/0/6	3/0/6
Group 2	6/0/6	5/0/6	5/1/6	5/4/6	4/4/4	1/1/1	0/0/0
Group 3	0/0/3	0/0/3	0/0/3	0/0/3	0/0/3	0/0/3	0/0/3
Group 4	0/0/3	0/0/3	0/0/3	0/0/3	0/2/3	0/2/3	0/0/0

Group 1: PCV2-infected/LPC-vaccinated/CSFV-challenged; group 2: PCV2-infected/CSFV-challenged; group 3: LPC-vaccinated/CSFV-challenged, and group 4: CSFV-challenged.

^a^The pigs were inoculated with 10^5.3 ^TCID_50 _of PCV2 (group 1 and 2), 1 dose of LPC vaccine (group 1 and 3), and 10^6.8 ^TCID_50 _of wild-type CSFV (ALD) (group 1, 2, 3, and 4) at 0, 12, and 27 dpi, respectively. The PCV2 and wild-type CSFV loads in saliva were detected by real-time PCR or reverse transcription real-time PCR, respectively.

^b^Number of PCV2-positive animals/number of wild-type CSFV-positive animals/number of surviving animals.

**Table 5:** Changes in the number of pigs showing viral shedding of porcine circovirus type 2 (PCV2) and wild-type classical swine fever virus (CSFV) in the feces of various treatment groups over time after CSFV challenge^a^.

dpi	27	30	33	36	39	42	45
Group 1	6/0/6^b^	6/0/6	6/0/6	6/2/6	6/3/6	6/1/6	6/0/6
Group 2	6/0/6	6/0/6	6/1/6	6/4/6	4/4/4	1/1/1	0/0/0
Group 3	0/0/3	0/0/3	0/0/3	0/0/3	0/0/3	0/0/3	0/0/3
Group 4	0/0/3	0/0/3	0/3/3	0/1/3	0/1/3	0/1/3	0/0/0

Group 1: PCV2-infected/LPC-vaccinated/CSFV-challenged; group 2: PCV2-infected/CSFV-challenged; group 3: LPC-vaccinated/CSFV-challenged, and group 4: CSFV-challenged.

^a^The pigs were inoculated with 10^5.3 ^TCID_50 _of PCV2 (group 1 and 2), 1 dose of LPC vaccine (group 1 and 3), and 10^6.8 ^TCID_50 _of wild-type CSFV (ALD) (group 1, 2, 3, and 4) at 0, 12, and 27 dpi, respectively. The PCV2 and wild-type CSFV loads in fecal samples were detected by real-time PCR or reverse transcription real-time PCR, respectively.

^b^Number of PCV2-positive animals/number of wild-type CSFV-positive animals/number of surviving animals.

### PCV2 infection decreased the levels of lymphocyte subgroups in LPC-vaccinated animals after CSFV challenge

**Figure 1:**
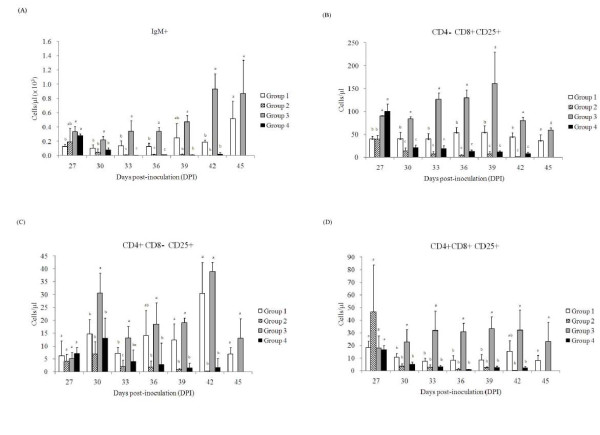
**Changes in the absolute number of IgM^+ ^(A), CD4^-^CD8^+^CD25^+ ^(B), CD4^+^CD8^-^CD25^+ ^(C), and CD4^+^CD8^+^CD25^+ ^(D) lymphocyte subsets in various treatment groups of pigs after classical swine fever virus (CSFV) challenged over time as determined by flow cytometry**. The pigs in group 1 (PCV2-infected/LPC-vaccinated/CSFV-challenged) and group 2 (PCV2-infected/CSFV-challenged) were inoculated with porcine circovirus type 2 (PCV2) at 0 dpi. The pigs in group 1 and group 3 (LPC-vaccinated/CSFV-challenged) were vaccinated with 1 dose of Lapinized Philippines Coronel (LPC) vaccine at 12 dpi. The pigs in all four groups were inoculated with wild-type CSFV (ALD strain) at 27 dpi. Data are shown as mean ± SD. ^a-d^Values with different superscripts indicate that the differences among groups are statistically significant (*P *< 0.05).

In order to understand how PCV2 affected the efficacy of LPC vaccine after wild-type CSFV challenge, changes in lymphocyte subsets among different groups were compared. Lymphocytes are classified by surface molecules of IgM, CD4, and CD8 into B-lymphocytes, T-helper lymphocytes, and cytotoxic T lymphocytes, respectively, and CD25 expression on lymphocytes is associated with the level of activation [14,15]. Thus, the populations of lymphocyte subsets of IgM^+^, CD4^+^CD8^-^CD25^+^, CD4^+^CD8^+^CD25^+^, and CD4^-^CD8^+^CD25^+ ^were measured in the present study. Although there was a transient change in the level of IgM^+^, CD4^+^CD8^-^CD25^+^, CD4^+^CD8^+^CD25^+^, and CD4^-^CD8^+^CD25^+ ^lymphocyte subsets after PCV2 infection and LPC vaccination (data not shown), a profound pattern of statistical difference between groups 1 and 3 was clear after wild-type CSFV challenge (Figure 1). PCV2 pre-infection could significantly decrease the cell proliferation of the four subsets stimulated by CSFV challenge. The numbers of IgM^+^, CD4^+^CD8^-^CD25^+^, CD4^+^CD8^+^CD25^+^, and CD4^-^CD8^+^CD25^+ ^lymphocyte subsets in group 1 were significantly lower than that of group 3 at 30-42 dpi, 30-42 dpi, 30-33 dpi, and 30-39 dpi, respectively. In the groups without LPC vaccination, the cells of all four subsets eventually gradually decreased over time after wild-type CSFV challenge and became undetectable at the end of the experiment.

### PCV2 infection caused transient delay of CSFV neutralizing antibody development

**Figure 2:**
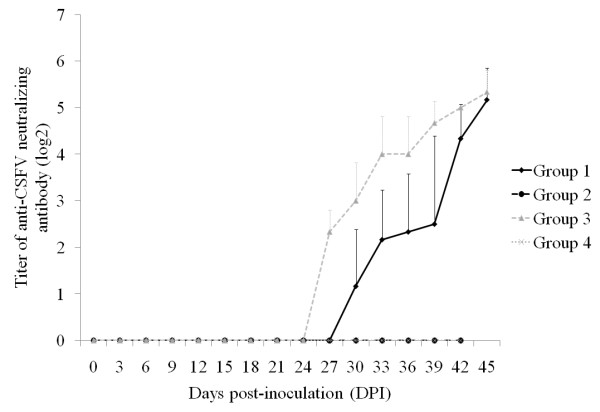
**The effect of porcine circovirus type 2 (PCV2) on the development of anti-classical swine fever virus (CSFV) neutralizing antibody in various treatment groups of pigs**. The pigs of group 1 (PCV2-infected/LPC-vaccinated/CSFV-challenged) and group 2 (PCV2-infected/CSFV-challenged) were inoculated with PCV2 at 0 dpi. The pigs of group 1 and group 3 (LPC-vaccinated/CSFV-challenged) were vaccinated with 1 dose of Lapinized Philippines Coronel (LPC) vaccine at 12 dpi. The pigs of all four groups were inoculated with CSFV (ALD strain) at 27 dpi. Data are shown as mean ± SD.

Based on the data, it is clear that PCV2 infection could result in an incomplete protection of LPC vaccine against wild-type CSFV challenge. Clinical signs, viremia, and viral shedding were observed in some of the LPC-vaccinated animals that were previously inoculated with PCV2. Compared to the group with LPC-vaccination alone, the lymphocyte subset related to antibody producing cells (IgM^+^) was also significantly decreased in the PCV2-infected and LPC-vaccinated group (Figure 1). In order to see if the decreased level in IgM^+ ^lymphocyte subgroup was correlated with the level of antibody production, the level of neutralizing antibody against CSFV was evaluated. In the group with LPC vaccination alone, the CSFV neutralizing antibody was detectable on the 15^th ^day after LPC vaccination and the level continuously increased after CSFV challenge till the end of the study. However, pre-infection of PCV2 delayed the onset of CSFV neutralizing antibody production (Figure 2), but the antibody level elevated after CSFV challenge and eventually reached a level similar to that of the simple LPC vaccination group (group 3) at the end of the study. The result indicates that PCV2 infection could transiently block the LPC vaccination-induced neutralizing antibody production. The anti-PCV2 antibody was only detected in PCV2-inoculated pigs (groups 1 and 2) between 15 and 45 dpi, and their levels were not significantly different (data not shown).

### PCV2 infection suppressed the development of CSFV-specific cell proliferative response

**Figure 3:**
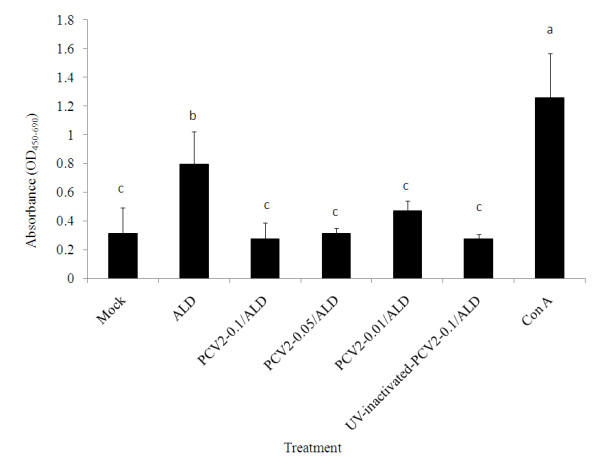
**The effect of porcine circovirus type 2 (PCV2) on the classical swine fever virus (CSFV)-specific cell proliferation reponse of peripheral blood mononuclear cells (PBMCs) in various ex vivo treatments**. The PBMCs were collected from pigs that had been vaccinated with Lapinized Philippines Coronel (LPC) vaccine twice, followed by challenge with wild-type CSFV (ALD) 6 weeks later. The PBMCs were pre-inoculated with 0.1, 0.05 or 0.01 MOI of PCV2, 0.1 MOI of UV-inactivated PCV2 or Con A at 5 μg/mL followed by inoculation with 1 MOI of wild-type CSFV 18 h later. This ex vivo CSFV-specific cell proliferation response was done in triplicates and assayed 4 days after wild-type CSFV stimulation by commercial kits as indicated in the material and methods. The values are shown as mean ± SD of five different pigs. ^a-d^Values with different superscripts indicate that the differences among groups are statistically significant (*P *< 0.05).

The CMI is a very important defensive system against CSFV infection, especially in the initial infection stage, when the neutralizing antibody is undetectable in the infected pigs [4,5]. In the present study, an ex vivo CSFV-specific cell proliferation experiment using PBMCs was performed to investigate whether PCV2 infection would interfere with the development of CMI against CSFV infection. A clear and significant CSFV-specific cell proliferation of PBMCs could be demonstrated four days after stimulation by the ALD strain of CSFV. However, the presence of PCV2 significantly reduced the cell proliferation response to the level as the mock control (Figure 3). The levels of PCV2-derived reduction among various doses of PCV2 were not statistically significant. Interestingly, the UV-inactivated PCV2 could also retain the inhibitory effect as the infectious virus did.

## Discussion

The present study clearly demonstrates that pre-existing PCV2 infection may affect the efficacy of LPC vaccine against wild-type CSFV infection. Without the pre-existing PCV2 infection, simple LPC vaccination could provide the pigs full protection from wild-type CSFV challenge with no fever and other clinical signs, viremia, and viral shedding (Tables 2, 3, 4, and 5). However, fever/clinical signs and transient viremia/viral shedding in saliva and feces could be noted in some and a majority of the pigs, respectively, pre-infected with PCV2 and then challenged with wild-type CSFV after LPC vaccination. These results indicate that the efficacy of LPC vaccine could be reduced by the presence of PCV2 infection. Clinically, the decreased efficacy of LPC vaccine may not cause any direct effect on the pigs; however, it may result in a shortcoming in the immune defensive system of the herd, which could allow the invasion of wild-type CSFV into the farm. The dissemination of the wild-type CSFV on the farm may induce latent infection or outbreak, depending on the status of the immunity in each herd. The decreased efficacy of the attenuated PRRSV vaccine by PCV2 has been documented [9]. PCV2 infection could interfere with the immune response against PRV, and the mechanism of this interference has also been investigated [10,11]. Nevertheless, information on the interaction between PCV2 and LPC vaccine is very limited. The results of the present study reveal a reduced efficacy of the LPC vaccine in the presence of PCV2. This information is important, especially in the area using attenuated vaccine to prevent and control CSFV.

The mechanism of reduced efficacy of LPC vaccine by PCV2 may correlate with the interference of activation and proliferation of lymphocyte subgroups, which include IgM^+^, CD4^+^, CD8^+^, and CD4^+^CD8^+ ^cells. These lymphocyte subgroups in pigs pre-infected with PCV2 and then challenged with CSFV after LPC vaccination (group 1) were significantly lower than those in pigs with LPC vaccination alone (group 3) (Figure 1). This PCV2-derived interference with lymphoycte activation reduced the activation of humoral immunity and CMI, and also correlated with the transiently delayed production of neutralizing antibody against CSFV and the inhibited CSFV-specific cell proliferation of PBMCs (Figures 2 and 3). According to previous studies, PCV2 may affect PRV recall antigen immune response through a soluble factor like IL-10 or structure component like CpG motif [10,11]. Attempts to investigate whether PCV2-derived inhibition in LPC vaccine-induced immune response is related to those soluble factors or components have also been carried out. The results showed that the CpG motif of PCV2 genome did not inhibit the CSFV-specific proliferation of PBMCs (data not shown). In addition, the levels of IL-10 in the plasma collected from the experimental animals and in the supernatant of the ex vivo CSFV-specific PBMC proliferation assay were not significantly different among various groups (data not shown). The data suggest that the mechanism of PCV2 interfering with the LPC vaccine-induced immune response may be different from how PCV2 affects the immune response induced by PRV. The interaction of PCV2 and the LPC strain of CSFV is worthy of further study.

Interestingly, although the LPC vaccine-induced immune response could be disturbed by PCV2, the level of antibody against PCV2 did not change after LPC vaccination or CSFV challenge. This result suggests that the deregulated immune response in the experimental animals is antigen-specific. The cell tropism of PCV2 and CSFV is overlapping, which includes monocyte-macrophage lineage cells and dendritic cells [7,16-19]. Although the impacts of PCV2 and CSFV on dendritic cells are different [16,19], the age of pig susceptible to the two viral infections is similar. Therefore, elucidation of the interaction between PCV2 and CSFV is very important in the control and prevention of both viral diseases.

The efficacy of all attenuated CSFV vaccines is correlated with the levels of humoral immunity and CMI induced by vaccination [4,5]. CMI is important when neutralizing antibodies are absent in pigs at the early stage of LPC vaccination [4,5]. The parameters of CSFV-specific CMI described in the literature, including interferon-gamma (IFN-γ) secretion cells, cell proliferative activity, and cytotoxicity of lymphocytes, were all correlated with the protection against CSFV infection [13,20,21]. In our study, CSFV-specific cell proliferation of PBMCs is a parameter of CMI. In the presence of PCV2, humoral and cell-mediated immune responses induced by LPC vaccine were both reduced. The level of neutralizing antibody was not reduced, but the onset of antibody production was sluggish; however, the CSFV-specific CMI was significantly reduced. The incomplete development of the immune response induced by LPC vaccine, including both humoral and cell-mediated immune responses, may have led to the temporal viremia and viral sheddding in the saliva and feces in the pigs following wild-type CSFV challenge.

The results of the ex vivo CSFV-specific PBMC proliferation assay might explain the mechanism of PCV2-derived reduction in the efficacy of LPC vaccine. Both live and UV-inactivated PCV2 suppressed the CSFV-specific PBMC proliferation. The cell population involved in the CSFV-specific PBMC proliferation included CD4^+^CD8^- ^and CD4^-^CD8^+ ^T lympocytes [22]. Significantly lower numbers of CD4^-^CD8^+^CD25^+^, CD4^+^CD8^-^CD25^+^, and CD4^+^CD8^+^CD25^+ ^were noted in the pigs pre-inoculated with PCV2. The PCV2-derived inhibition in the CSFV-specific cell proliferative response of PBMCs was correlated to the lymphocyte subset change after CSFV challenge. The number and function of T helper and cytotoxic T lymphocytes against CSFV may also be depressed by PCV2-derived inhibition. IL-10 and CpG motifs have been demonstrated to be PCV2-associated immunosuppressing factors in the PRV model [10,11]; however, neither factor was illustrated in the inhibition on CSFV-specific PBMC proliferation. The present study demonstrates that UV-inactivated PCV2 could inhibit CSFV-specific PBMC proliferation, and a similar inhibition pattern was also observed in the PRV model [11]. The surface of PCV2 virion is composed of capsid protein, which binds to heparin sulfate and chondroitin sulfate B glycosaminoglycan receptors on the cell membrane during the infection process [23]. It is speculated that the PCV2 capsid protein could be a PCV2-associated immunosuppressing factor; this factor is therefore worth further study.

In contrast to pigs with simple CSFV challenge, pigs pre-inoculated with PCV2 and then challenged with CSFV showed significantly shortened survival times and more severe clinical signs. The results suggest that PCV2 may modulate the pig immune system leading pigs to become more susceptible to CSFV infection. It is known that CSFV is able to induce lymphocyte apoptosis and necrosis, directly or indirectly [16,24]. In addition, PCV2 induces lymphoid depletion in PMWS-affected pigs [7,25] and reduces the function of macrophages such that the phagocytosis and microbicidal capability of alveolar macrophages are decreased [26]. How PCV2 accelerates the severity of CSFV infection is worth further study.

The present study has demonstrated that PCV2 could decrease the efficacy of LPC vaccine. This information is important, especially in the CSFV endemic areas using attenuated vaccine for CSFV control and prevention. Several measures should be considered to ameliorate the problem caused by pandemic PCV2 infection such as modification of the CSFV vaccination schedule or the use of a vaccine booster. Finally, whether the newly developed CSFV subunit vaccines such as the E2 vaccine are resistant to interference by PCV2 is another important issue to be clarified.

## Competing interests

The authors declare that they have no competing interests.

## Authors' contributions

YLH participated in the design of the study, carried out the animal experiment, analysed the results, and prepared the manuscript draft. CML, YCT, and MYC carried out the immunoassay. MCD carried out the animal experiment. CYC carried out the virological analyses. VFP and CRJ engaged in the design and coordination of the study and interpretation of the results as well as preparation of the manuscript. All authors read and approved the final manuscript.
